# The BELFRAIL (BF_C80+_) study: a population-based prospective cohort study of the very elderly in Belgium

**DOI:** 10.1186/1471-2318-10-39

**Published:** 2010-06-17

**Authors:** Bert Vaes, Agnes Pasquet, Pierre Wallemacq, Nawel Rezzoug, Hassan Mekouar, Pierre-Alexandre Olivier, Delphine Legrand, Catharina Matheï, Gijs Van Pottelbergh, Jan Degryse

**Affiliations:** 1 Institute of Health and Society, Université Catholique de Louvain, Clos Chapelle-aux-Champs 30, bte 3005, 1200 Brussels, Belgium; 2 Department of Cardiology, Cliniques Universitaire St-Luc, Université Catholique de Louvain, Brussels, Belgium; 3 Laboratory of Analytical Biochemistry, Cliniques Universitaires St Luc, Université Catholique de Louvain, Brussels, Belgium; 4 Department of Primary Health Care, Katholieke Universiteit Leuven, Leuven, Belgium

## Abstract

**Background:**

In coming decades the proportion of very elderly people living in the Western world will dramatically increase. This forthcoming "grey epidemic" will lead to an explosion of chronic diseases. In order to anticipate booming health care expenditures and to assure that social security is funded in the future, research focusing on the relationship between chronic diseases, frailty and disability is needed. The general aim of the BELFRAIL cohort study (BF_C80+) _is to study the dynamic interaction between health, frailty and disability in a multi-system approach focusing on cardiac dysfunction and chronic heart failure, lung function, sarcopenia, renal insufficiency and immunosenescence.

**Methods/Design:**

The BF_C80+ _is a prospective, observational, population-based cohort study of subjects aged 80 years and older in three well-circumscribed areas of Belgium. In total, 29 general practitioner (GP) centres were asked to include patients aged 80 and older. Only three exclusion criteria were used: severe dementia, in palliative care and medical emergency. Two sampling methods for the recruitment of patients were used. Between November 2, 2008 and September 15, 2009, 567 subjects were included in the BF_C80+ _study. Every study participant was invited to undergo four study visits. The GP recorded background variables and medical history and performed a detailed anamnesis and clinical examination. The clinical research assistant performed an extensive examination including performance testing, questionnaires and technical examinations. Echocardiography was performed at home by a cardiologist. A blood sample was collected in the morning. Follow-up reporting of hard outcome measures including mortality, hospitalization and morbidity was organized. A second data collection is planned after 18 months.

**Discussion:**

The BF_C80+ _was designed to acquire a better understanding of the epidemiology and pathophysiology of chronic diseases in the very elderly and to study the dynamic interaction between health, frailty and disability in a multi-system approach. The wide variety of dimensions investigated in the BF_C80+ _will enable us not only to investigate in depth the relationship between the different physiological systems but also to initiate new research questions based on this unique database of community-dwelling elderly.

## Background

Health care and social security systems in industrialized countries are confronted with ageing populations. By 2050, 10% of people living in Belgium will have reached the age of 80 or older [1]. This forthcoming "grey epidemic" will lead to an explosion of chronic diseases. This situation has stimulated researchers to focus their efforts on studying relationships between chronic diseases, frailty and the development of disability [2,3]. Fried et al. [4] have clearly shown that there is an overlap between chronic diseases, frailty and disability. In this regard the concept of frailty fulfils an important role. Although there are a large variety of models, definitions and criteria, a consensus view exists that considers frailty as a multidimensional geriatric syndrome with biological, physiological and psychosocial components, and as a state of increasing vulnerability and loss of adaptability to stress [5,6]. Campbell and Buchner [7] described frailty as a condition or syndrome which results from a multi-system reduction in reserve capacity to the extent that a number of physiological systems are close to, or past, the threshold of symptomatic clinical failure. A better understanding of the dynamic process leading from health to frailty and eventually disability is of primary interest if preventive interventions are planned.

In Belgium general practitioners (GPs) have a prominent place in the health care system. More than 95% of the population is reported to consult the same GP or practice (duo or group) in case of health problems. More than 90% of people aged 65 and older have at least one contact with their GP every year, with an average of 11.9 contacts per year at the age of 75 and older [8].

The general aim of the BELFRAIL cohort study is to acquire a better understanding of the epidemiology and pathophysiology of chronic diseases in the very elderly and to study the dynamic interaction between health, frailty and disability in a multi-system approach. Therefore the BF_C80+ _focuses on different systems; (1) cardiac dysfunction and chronic heart failure, (2) lung function, (3) sarcopenia, (4) renal insufficiency and (5) immunosenescence and the profile of immune risk.

### Cardiac dysfunction and chronic heart failure

In our ageing society the burden of chronic heart failure is rising. The prevalence increases with age from 0.7% in people aged 55-64 years to 2.7% in those aged 65-74 years and 13.0% in those aged 75-84 years [9]. Heart failure not only has negative consequences for functional status and well-being but also leads to increased mortality [9]. However, diagnosing chronic heart failure is notoriously difficult, especially in the elderly who often have multiple comorbidities and may present with many other possible causes for dyspnoea, fatigue or peripheral oedema. With increasing average patient age, primary care physicians will become increasingly important as the principal diagnosticians and treating physicians. In this setting, poor availability of routine echocardiography leads to considerable over- and under-diagnosis of heart failure [10,11]. This emphasizes the need for a simple test, easily applicable in primary care settings, to identify elderly patients at risk and to initiate timely treatment to reduce mortality and improve quality of life. Over the last decade, brain natriuretic peptide (BNP) and its amino-terminal portion N-terminal pro-brain natriuretic peptide (NT-proBNP) have been extensively studied. However, a recent systematic review found limited evidence for the usefulness of natriuretic peptide measurement for the diagnosis of cardiac dysfunction or heart failure in community-dwelling elderly patients aged 75 and over and concluded that important questions about the implementation of the natriuretic peptide test in daily practice remain unsolved [12]. The BF_C80+ _was conceived (1) to examine further the diagnostic accuracy of natriuretic peptides for cardiac dysfunction in elderly people in the community and (2) to develop an algorithm, easily applicable in primary care, for the diagnosis of chronic heart failure in the very elderly by estimating the added value of natriuretic peptides and ECG beyond history taking and clinical examination.

### Lung function

With regard to the burden of respiratory diseases in the elderly, many fundamental issues remain unresolved. Few reliable data are available concerning the prevalence of chronic obstructive pulmonary disease (COPD) and asthma [13], and the global initiative for chronic obstructive lung disease (GOLD) criteria [14] for the diagnosis of COPD have been debated and appear not to be meaningful in the elderly [15]. Moreover, the lack of clear reference data for the very elderly makes assessment of pulmonary function a real challenge [16,17]. Predictive models for spirometric variables have been proposed but are based on very small samples that are rarely representative of the population in an area [18]. Two aims of the BF_C80+ _study are (1) to collect respiratory reference data from a large sample of the elderly population in Belgium in order to develop an adapted predictive model (regression equation that allows computation of predicted values) and (2) to gain better insight into the prevalence of respiratory symptoms and obstructive lung disease in octogenarians. Clustering with other chronic diseases will also be studied, and the ability of older patients to perform good-quality spirometric tests will be investigated.

### Sarcopenia

Sarcopenia has been described as an age-related decline in muscle mass in older people [19]. Current definitions of sarcopenia include both a loss of muscle strength and a decline in functional quality in addition to the loss of muscle protein mass [20]. However, it is unclear whether a decline in functional capacity results from the loss of muscle mass and/or the qualitative impairment of the muscle tissue [21]. The age-associated changes in muscle mass explain less than 5% of the variance in the change in strength with ageing [20]. Sarcopenia is associated with many adverse outcomes like increases in morbidity, falls, institutionalization and onset of disability [22]. It has been suggested that the contribution of muscle mass to certain outcomes may be primarily because of its association with muscle strength [20]. Decreased strength, most often grip strength, has been identified as an important sign of frailty [23]. Grip strength appears to be a robust predictor of functional decline, disability and mortality [19]. In the BF_C80+ _the relationship between grip strength, muscle mass and clinical outcome will be studied. Also the relationship with nutritional status, performance tests and biological indicators will be investigated.

### Renal insufficiency

Chronic kidney disease (CKD) is increasingly recognized as an important problem in public health because of its high prevalence [24] and its association with increasing mortality, cardiovascular events and hospitalizations [25]. "Gold standard tests" based on ethylenediaminetetraacetic acid (EDTA), inulin, iohexol or diethylene triamine pentaacetic acid (DTPA) clearance that aim to measure the glomerular filtration rate (GFR) are difficult to perform in clinical practice. Cockcroft and Gault [26] developed a method to estimate renal function based on the serum creatinine value and the patient's body weight and age. Since then, many researchers have tried to find a better formula based on serum creatinine or cystatin C [27]. However, none of these methods has been validated in a large population of community-dwelling elderly patients. The aims of the BF_C80+ _are, first, to achieve a better understanding of the prevalence, complications and evolution of CKD in elderly patients, and second, to identify the most accurate existing method or to develop a new method, applicable in clinical practice, to estimate the GFR in elderly patients.

### Immunosenescence and the immune risk profile

Deterioration of the immune system with ageing, referred to as immunosenescence, is believed to be a prominent pathophysiological feature of frailty [28]. The hallmarks of immunosenescence are a decrease in adaptive immunity and increased low-grade chronic inflammatory status, which has been referred to as inflamm-ageing. The former results in a decreased ability to effectively control infectious diseases and a generally poor response to vaccination, while inflamm-ageing seems to underlie most of the age-related diseases (e.g. atherosclerosis, dementia, sarcopenia, diabetes, etc.) and has been shown to be related to mortality of all causes in older persons [29,30]. The chronological age at which immunosenescence becomes clinically important is most likely influenced by many factors, including the pathogen load to which individuals are exposed throughout life. There is considerable evidence that human cytomegalovirus (CMV) in particular plays an important role in immune modulation later in life [31]. Understanding how and why immune responsiveness changes in humans as they age is essential for developing strategies to prevent or restore dysregulated immunity and assure healthy aging. Therefore, we will explore the complex relationship between immunosenescence and frailty, disease and death in the BF_C80+_. Furthermore, we will investigate the role of CMV and other pathogens in the process of immunosenescence and the mechanisms underlying it.

## Methods

### Study design and setting

The BF_C80+ _is a prospective, observational, population-based cohort study of subjects aged 80 years and older in three well-circumscribed areas in Belgium. The study protocol was approved by the Biomedical Ethics Committee of the Medical School of the Université Catholique de Louvain (UCL) of Brussels, Belgium (B40320084685).

Sample size calculations were based on the first focus of this study: to determine the diagnostic accuracy of clinical signs and symptoms for heart failure. Therefore the sample size calculation was based on the following premises: 1) the expected prevalence of "cardinal" symptoms (i.e. dyspnoea, fatigue, peripheral oedema) of CHF in octogenarians of 30%, 2) the worst-case scenario in which none of these criteria would have any diagnostic value (i.e. proportion of any symptom of 0,5) and taking into account an acceptable confidence interval of 90%, and 3) assuming a prevalence of CHF of 15% in octogenarians. The sample size for the BF_C80+ _was estimated on 420 subjects. Taking into account a refusal rate of not more than 10% and a yearly mortality rate of 10% a sample size of 550 was proposed.

General practitioners in three Belgian areas were invited to participate in the study. In Wallony (région de Dinant), Brussels and Flanders (Druivenstreek), respectively, 17, 2 and 10 GP centres agreed to participate. Participating centres were invited to a training meeting in order to standardize the recording of the medical history, anamnesis and clinical examination. The GP centres received study information with a detailed study protocol and a CD-ROM with visual and audio examples of the clinical examination and recent guidelines on heart failure and hypertension.

The participating centres were asked to include patients aged 80 and older in the cohort. Only three exclusion criteria were used: (1) dementia (known mini-mental state examination (MMSE) < 15/30), (2) in palliative care and (3) medical emergency. Two sampling methods for the recruitment of patients were used. Two GP centres were asked to include all eligible patients. The remaining 27 centres were asked to include a maximum of three consecutive patients during a three-week interval. In these three weeks the GP also planned his visit. This interval was repeated five times so every participating centre included a maximum of 15 patients. Every interval of recruitment was started on a different day to avoid selection bias.

On November 2, 2008 the inclusion period was started in Wallony. All participating centres in this area recruited patients according to the second sampling method, thus a maximum of 15 patients. On February 28, 2009 inclusion was stopped in Wallony. On March 16, 2009 the inclusion period was started in Flanders and Brussels. One centre in Flanders and one centre in Brussels recruited all eligible patients. The remaining centres (Flanders (n = 9), Brussels (n = 1)) recruited patients according to the second sampling method. On September 15, 2009 inclusion was stopped in Flanders and Brussels.

### Visit to general practitioner

Eligible patients were first flagged by the GP and received an information letter about the study. After providing informed consent, patients entered the study population. In the weeks after flagging, the GP recorded the social situation, medical history and medication for each patient and undertook a detailed anamnesis and clinical examination.

The social anamnesis consisted of questions about ethnic group, marital status, type of living (at home or institutionalized), level of education and professional history. The GP was asked to record important antecedents in the medical history of the study subjects and to list the medical problems for which the subject was currently followed. Afterwards, a detailed recording of non-cardiovascular and cardiovascular comorbidities took place.

Non-cardiovascular morbidities were defined as a positive response from the GP about the presence of thyroid problems, anaemia, asthma, COPD, Parkinson's disease, arthritis, osteoarthritis, documented osteoporosis, malignancies, depression and renal insufficiency. A distinction was made between an active and cured pathology. Active status was defined as being present (even if well controlled by treatment) or having occurred less than six months ago. The GP was asked whether the subject had had a knee or hip replacement or had undergone important surgery less than or more than 12 months ago. Tetanus, pneumococcal and influenza vaccination status was registered.

Cardiovascular morbidities were defined as a positive response for the presence of hypertension, diabetes mellitus, hyperlipidaemia, history of angor pectoris or myocardial infarction, known cardiomyopathy, history of transient ischaemic attack (TIA) or cerebro-vascular accident (CVA), peripheral arterial disease, history of decompensated heart failure, atrial fibrillation, valvular disease or history of oedema of the lower extremities. If present, details were requested about the date of diagnosis, the diagnostic pathway and nature of treatment. The GP was asked to report important cardiovascular intervention or surgery (percutaneous transluminal coronary angioplasty (PTCA) or stenting, coronary, valvular or arterial surgery, or the placement of cardiac device) less than or more than 12 months ago.

Current medication was recorded by the GP.

A structured and standardized anamnesis was performed and included questions about symptoms of angina pectoris (at rest or during exercise), dyspnoea (Medical Research Council (MRC) scale), fatigue, orthopnoea, cough (nocturnal or diurnal), wheezing, oedema of the lower extremities, palpitations, loss of appetite, smoking status, alcohol intake, recent changes in symptoms, recent weight change and alteration of mental and psychological status.

The clinical examination consisted of a blood pressure measurement in the sitting position on both arms that was repeated after 2 min. The GP used his or her own blood pressure meter. Heart rate (with auscultation over 30 s, × 2), heart rhythm (regular or irregular) and breathing rate at rest (with observation over 30 s, × 2) were measured. Heart auscultation was performed to detect an S3, S4 or cardiac murmur (systolic or diastolic). The apex beat was palpated with the patient in a supine and lateral position, and if present the GP was asked to describe whether it was displaced (outside the mid-clavicular line in supine position), enlarged (> 2 fingers) or sustained (in left lateral position). Lung auscultation was performed to detect crepitations, wheezing and rhonchi. The carotid arteries were auscultated to detect a murmur and the peripheral arteries (femoral, popliteal and dorsal and tibial arteries) were palpated. The jugular venous pressure (JVP) was measured in a 45° reclining position and noted if raised (> 3 cm between the angle of Louis (manubrio-sternal joint) and the highest level of jugular (internal or external) vein pulsation). In the same position, the presence of hepatojugular reflux was checked for. Hepatojugular reflux was present when the vertical height of the JVP increased by more than 1 cm and remained elevated for as long (15-20 s) as pressure was applied to the epigastrium. The GP was asked to determine whether hepatomegaly or oedema of the lower extremities was present and if he or she had a suspicion of ascites.

Finally, the GP was asked whether, in his or her opinion, the patient suffered from chronic heart failure, and to determine his or her degree of certainty (2, 10, 25, 50, 75, 90 or 98% certain). The reason why the GP thought chronic heart failure was or was not present was noted and the New York Heart Association (NYHA) class scored for those patients with a suspicion of chronic heart failure.

The practice in Flanders that included all eligible patients also determined an ankle-brachial index with the patient in supine position with oscillometric pressure measurements using a standard automated blood pressure cuff system (SCVL-2007, Diegel Healthworks^®^, Auckland, New Zealand).

### Visit to clinical research assistant

After inclusion of a patient, the data manager made an appointment for the clinical research assistant (CRA) to visit the patient. Three CRAs were trained to perform an extensive examination with performance testing, questionnaires and technical examinations. The French-speaking patients were visited by a French-speaking CRA (n = 1) and the Dutch-speaking patients by a Dutch-speaking CRA (n = 2). This visit lasted between 90 and 150 minutes depending on the general state of the patient. The following tests and questionnaires were performed by the CRA:

- Biometry: circumference of upper arm, calf and upper leg, skin fold of upper arm, calf and upper leg, weight, height, span width and knee height.

- Blood pressure: electronically measured (Omron 705IT^®^, Omron, Japan) in sitting position on both arms and repeated after 2 min.

- Vision: asking the respondent "are you able to recognize someone's face at a distance of four metres?" [32].

- Hearing: asking the respondent "are you able to follow a conversation with one and four persons?" both with a hearing aid if needed [32].

- Incontinence: asking the respondent whether he or she lost urine unintentionally [33].

- Performance testing: the performance-based tests of physical function included timed measures of walking speed, rising from a chair, putting on and taking off a cardigan, and maintaining balance in a tandem stand. The performance tests have been used in several studies and have been shown to be a reliable and valid measure of physical functioning [34,35]. For the walking test, respondents were asked to walk 3 metres, turn around, and walk back the 3 metres as quickly as possible. For the chair-stand test, respondents were asked to fold their arms across their chest and to stand up from a sitting position and sit down five times as quickly as possible. For the cardigan test, respondents were asked to put on and take off the cardigan. For the ability to maintain balance in tandem stand the respondent was asked to put the heel of one foot in front of the other and to stand still as long as possible.

Categories of performance were created for each set of performance measures to permit analyses that included those unable to perform a task. For the walking test, chair-stand test and cardigan test, those who could not complete the task were assigned a score of 0. Those completing the task were assigned scores of 1 to 4, corresponding to the quartiles of time needed to complete the task, with the fastest time scored as 4. For the balance in tandem stand a score of 0 was assigned to those who were unable to perform the test or maintained the tandem stand for less than 3 seconds (< 3 s). For those maintaining a tandem stand for more than 3 seconds but less than 10 seconds (3-9.99 s) a score of 1 was assigned and for those maintaining the tandem stand for 10 seconds or more a score of 2. A summary performance scale, ranging from 0 to 14, was created by summing the category scores.

- Activities of daily living (ADL): functional limitations were assessed by asking the respondent to describe the degree of difficulty they had with six activities of daily living (ADL): climbing stairs, walking 5 min outdoors without resting, getting up and sitting down in a chair, dressing and undressing oneself, using own or public transportation, and cutting one's own toenails [36-38]. Response categories ranged from (1) "No I cannot" to (5) "Yes without difficulty." The total score was calculated by summing the scores of all activities and ranged between 6 and 30.

- LASA Physical Activity Questionnaire (LAPAQ) [39]: the LAPAQ is a face-to-face questionnaire http://www.lasa-vu.nl that covers the frequency and duration of walking outside, bicycling, gardening, light household activities, heavy household activities, and a maximum of two sport activities during the previous two weeks. Walking and bicycling for transportation purposes are considered as common daily activities, and not as sport activities. The duration of the activities assessed with the LAPAQ was categorized into six groups. The duration of the activities, 0, 1-15, 16-30, 31-60, 61-120 and > 120 min per day, were assigned scores 0, 1, 2, 3, 4 and 5 (called the duration score), respectively. The total score of the LAPAQ for each activity in two weeks was calculated by multiplying the frequency of the activity by the duration score. For instance, eight walking sessions of 45 min each in two weeks corresponded to eight × duration score 3 = 24. If the activity was performed several times per day, the total duration of that activity per day was calculated. For example, 45 walking sessions of 10 min each in two weeks corresponded to (45 × 10)/14 = 32 min of walking per day. In this example the score for walking was 14 × duration score 3 = 42. The total activity was calculated by summing the scores of the individual activities over two weeks.

- Geriatric Depression Scale (GDS-15): the 15-item Geriatric Depression Scale (GDS-15) is the shortened, less time-consuming version of the 30-item GDS which has been especially designed to screen for depression in the elderly [40]. Both the long and the short form of the GDS focus on functional and mood symptoms of depression rather than on potentially misleading somatic features. Several studies have validated the use of the GDS-15 for screening for depressive disorders in old age in geriatric inpatients [41], outpatients [42] and in primary care [43]. It has been shown that the GDS-15 has a good accuracy in screening for depression in the community-living very elderly [44].

- MMSE [45]: cognitive function was assessed by the MMSE, with scores ranging from 0 to 30 points (optimal). The test evaluates in a global way cognitive efficiency by examining orientation in time and space, short- and middle-term memory, calculation, comprehension and constructive praxis. Any score greater than or equal to 25 points is effectively normal (intact). Below this, scores can indicate severe (≤ 9 points), moderate (10-20 points) or mild (21-24 points) deficits [46]. The raw score may also need to be corrected for educational attainment and age.

- Barthel Index [47]: the Barthel Index consists of 10 items that measure a person's daily functioning, specifically the activities of daily living and mobility. The items include feeding, moving from chair to bed and return, grooming, transferring to and from a toilet, bathing, walking on a level surface, going up and down stairs, dressing, and continence of bowels and bladder. The assessment can be used to determine a baseline level of functioning and can be used to monitor improvement in activities of daily living over time. The items are weighted according to a scheme developed by the authors [47]. The person receives a score based on whether they have received help while doing the task. The scores for each of the items are summed to create a total score, with a maximum score of 100. The higher the score the more "independent" the person. Independence means that the person needs no assistance for any part of the task.

- Tinetti test [48]: the Tinetti test comprises a series of tests that measures subjects' gait and balance in order to estimate the risk of falls. Subjects were scored 0 to 2 on the individual items, with 0 representing the most impairment and 2 representing independence. The individual items were combined into three measures: (1) an overall gait score (12 points), (2) an overall balance score (16 points) and (3) a gait and balance score (28 points). Subjects who scored 25 to 28 were classified as at low risk of falls. Those who scored 19 to 24 points were classified as at moderate risk of falls and those who scored 18 or below were classified as at high risk of falls.

- IADL-E Lawton: the ability of subjects to perform instrumental activities of daily living (IADL-E) was measured using the scale developed by Lawton and Brody [49]. The instrument consists of eight items (ability to use telephone, shopping, food preparation, housekeeping, laundry, mode of transportation, responsibility for own medications and ability to handle finances) and has been widely accepted as a valid and reliable measure for use in elderly community populations. One item was added, namely the ability to repair and maintain the house. The scale was completed by the CRA during a home visit using the "best available" information, which included self-report, CRA observation, or proxy report. Responses were recorded for each item. To improve the sensitivity of the scale, the trial altered the scoring of each item from the original two-point scale to scores which ranged from 1 to 4. Scores increased with level of dependence, and the same scoring system was used for both sexes. If it was impossible to score an item, this item was excluded from the overall score. Scores ranged from 9/36 (independent) to 36/36 (complete dependency).

- IADL (Katz-score): the Katz Index of Activities of Daily Living is an assessment tool that is frequently used with the geriatric population in many settings, including patients' homes [50]. It is used to measure function and how it changes over time in older people who have chronic illnesses. The Katz scale considers six basic domains: bathing, dressing, toileting, ambulating or transferring, continence and feeding. For each of the six function domains, five possible function levels are scored ranging from complete independence (6/30) to complete dependence (30/30).

- The Groningen Frailty Indicator (GFI): this is a short 15-item questionnaire aiming to identify patients that have diminished reserves in one or more of the core domains of functioning [51,52]. These domains are: mobility, physical fitness, comorbidity, weight loss, vision, hearing, cognition and psychosocial resources. It has empirically been shown that all these domains are associated with a variety of adverse outcomes. The current view on the clinical measurement of frailty encompasses the whole person's functioning and physiology, emphasizing the interaction of physical and psychosocial systems [53]. The GFI builds on the assumption that strong interaction effects often exist between different weaknesses of older patients, reinforcing each other into a downward spiral of further decline.

- Sense of coherence: the concept of sense of coherence (SOC) was put forward by Aaron Antonovsky in 1979 to explain why some people become ill under stress and others stay healthy [54-56]. It arose from the salutogenic approach, that is, the search for the origins of health rather than the causes of disease. The SOC is defined as: "The extent to which one has a pervasive, enduring though dynamic, feeling of confidence that one's environment is predictable and that things will work out as well as can reasonably be expected." In other words, it is a mixture of optimism and control. It has three components: comprehensibility, manageability and meaningfulness. Comprehensibility is the extent to which events are perceived as making logical sense, that they are ordered, consistent and structured. Manageability is the extent to which a person feels they can cope. Meaningfulness is how much one feels that life makes sense and challenges are worthy of commitment. For this study the 13-item scale was used. Every item was scored with a Likert scale ranging from 1 to 7, generating a total scale range from 13-91, a high score representing a strong SOC. Before calculating the total score, five items were reversed according to the original codebook as presented by Antonovsky [57]. The division into a low and high/medium group was dichotomized at the lower tertile.

- Locus of Control (LOC): locus of control refers to the extent to which individuals believe that they can control events that affect them. Individuals with a high internal locus of control believe that events result primarily from their own behaviour and actions. Those with a high external locus of control believe that powerful others, fate, or chance primarily determine events. The Multidimensional Health Locus of Control (MHLC) Form A, developed by Wallston and Wallston in 1978, was used [58]. The MHLC, which is a six-point Likert scale, contains 18 questions classified into three subscales: Internal HLC, Powerful-Others HLC, and Chance HLC. Each subscale contains six questions. For each question subjects chose one out of six answers ranging from "strongly agree" to "strongly disagree".

- Grip strength: grip strength was measured in the dominant hand using a JAMAR^® ^Plus digital handheld dynamometer. Three attempts at maximal squeeze were recorded.

- Basic spirometry: this was performed using a Spirobank spirometer (MIR, Rome, Italy). This is a hand-held instrument for lung function tests that has been wired into a computer. Winspiro software (MIR) compares the measured values with reference tables and automatically calculates the reproducibility of the performed spirometry in accordance with European Respiratory Society guidelines [59]. The CRA enthusiastically demonstrated the correct manoeuvres before the patient attempted them. The minimum number of forced vital capacity (FVC) manoeuvres required to meet quality goals is three, but since older patients require an average of five, up to eight manoeuvres were performed when needed in order to meet the goals. When patients appeared to be exhausted the test was interrupted.

- Electrocardiogram (ECG): a 12-lead ECG was recorded on a QRS Universal ECG device (QRS Diagnostic, Plymouth, USA, http://www.qrsdiagnostics.com. QRS medical devices are manufactured under an ISO 13485 Registered Quality Management System. The Universal ECG device is FDA approved and CE Marked in accordance with medical device directive (MDD) 93/42/EEC. An automated protocol was produced by the QRS Diagnostic software based on the "Louvain" diagnostic algorithm [60]. Each ECG was digitally stored and analysed off-line by an experienced cardiologist according to the Minnesota Code Classification System for Electrocardiographic Findings.

- Bio-electrical impedance (BIA): BIA is based on the relationship between the volume of a conductor and its electrical resistance. We used BIA mainly to estimate skeletal muscle mass [61]. Validated formulas appropriate for assessment of fat-free mass (FFM) and skeletal muscle mass (SMM) in the elderly were used as suggested by Duerenberg et al. [62]. The BODYSTAT 1500 MDD device (Bodystat LTD, Douglas, Isle of Man, UK) was used and dual-frequency measurements were performed with patients in a supine position. Patients wearing a pacemaker were excluded from the procedure.

- Exhaled NO: Nitric oxide (NO) is detectable in the exhaled air of humans. An increase in the concentration of exhaled nitric oxide (eNO) has been found in asthmatic patients including those with mild disease [63]. Levels of eNO parallel the inflammatory process in the asthmatic airway [64]. Recommendations for standardized procedures for measurements of exhaled lower respiratory nitric oxide have been published by the American Thoracic Society [65]. The NIOX MINO portable device (Aerocrine, Solna, Sweden, http://www.aerocrine.com was used. The device produces an auditory and visual feedback signal to guide patients. Patients were encouraged to perform a 10-second exhalation manoeuvre. When this appeared difficult, a 6-second procedure was accepted.

### Echocardiography

Echocardiograms were performed at home using a commercially available portable system (CX50, Philips, Andover, Massachusetts, USA) with M-mode, 2-dimensional, and pulsed, continuous-wave and colour-flow Doppler capabilities. Echocardiograms were performed by a cardiology fellow fully trained in echocardiography. All patients were examined in left lateral decubitus. A complete examination comprising standard parasternal short and long axis, apical and subcostal 2D views was performed according to the recommendation of the American and European society of Echocardiography [66]. Colour, pulsed and continuous Doppler were used to assess the presence and severity of valvular lesions. Diastolic function was assessed using mitral flow velocities obtained by pulsed Doppler or pulsed tissue Doppler at the level of the mitral annulus. Additional apical and parasternal views for assessment of tissue velocity (colour tissue Doppler) and deformation (2D strain or speckle tracking) were also recorded. Still frames for M-Mode, continuous and pulsed Doppler and cineloops for assessment of left ventricular (LV) function were digitally stored on DVD and later transferred to a workstation. All the measurements were performed off-line using Xcelera software (Philips, Andover, Massachusetts, USA). LV function was calculated by the Simpson biplane method. Tissue velocity and deformation were analysed using Q lab software (Philips, Andover, Massachusetts, USA).

### Laboratory tests

Several blood specimens were collected on each patient after fasting (between 7.00 AM and 10.30 AM), and immediately stored in a refrigerated container until arrival in the central laboratory (< 3 h after blood collection). Plasma (EDTA, heparin) or serum samples were obtained after centrifugation. They were separated as aliquots (n: 4-6, depending on the volume obtained) and immediately stored frozen at -80°C until analysis. The analytical process was organized to avoid several freeze-thaw cycles. The biomarkers investigated included a wide range of cardiac, renal, thyroïd, bone metabolism, nutritional and inflammatory biomarkers, such as BNP and NTpro-BNP, TSH and free T4, creatinine and cystatin C, HDL- and LDL-cholesterol, total-cholesterol and triglycerides, hemoglobin, calcium, 25-hydroxyvitamin D, parthyroid hormone, phosphorus, transthyretin (prealbumin), IGF1, osteocalcin, usCRP, CMV IgG, a series of trace elements (including aluminium, zinc, copper, selenium, manganese, nickel, etc.), Clara Cell proteins (CC16) and surfactant protein D (SPD). A large panel of inflammatory biomarkers and cytokines (12 Cytokines Array 1) was analyzed using Biochip arrays on the Evidence Investigator™ analyzer (Randox Laboratories Limited, Crumlin, UK). The combination of immobilised ligands specific to different bio-markers, chemiluminescent immunoassays and charged coupled device camera enables to analyze simultaneously the 12 cytokines using a limited volume of serum (100 μL).

All measurements were performed in the laboratories of the Cliniques universitaires St Luc, Brussels, except the CC16 and SPD assays, determined in the laboratory of the Louvain centre for Toxicology and Applied Pharmacology (LTAP, Université catholique de Louvain, Medical School, Brussels) in accordance with strict quality controls programmes and proficiency testing schemes. The UniCel^® ^Dxl 800 Immunoassay System (Beckman-Coulter, Brea, USA) was used to determine the BNP, Parathyroid hormone (PTH), TSH, and free T4 concentrations; the Dade-Dimension^® ^Xpand (Siemens, Deerfield, USA) was used to measure the NTpro-BNP concentration (NTpBNP); the UniCel^® ^DxC 800 Synchron (Beckman-Coulter, Brea, USA) was used to measure creatinine, HDL- and LDL-cholestserol, Total-cholesterol, triglycerides, calcium, phosphorus, transthyretin, usCRP, and cystatin C. The Sysmex XE-2100 automated hematology analyser (Milton Keynes, UK) was used to measure the hemoglobin concentrations. The CMV IgG concentrations were measured on the ARCHITECT^® ^i4000SR (Abbott Diagn, Abbott Park, Illinois, USA). The 25-OH-Vitamin D, IGF1, and osteocalcin were measured on the LIAISON^® ^(Diasorin, Saluggia, Italy). Twenty three trace metal elements were simultaneously measured by Inductively Coupled Plasma Mass Spectrometry (ICP-MS), on a 7500 Series ICP-MS system (Agilent Technologies, Santa Clara, USA). CC16 and SPD were determined by ELISA using a Tecan plateform (Männedorf, Switzerland) and reagents kits from BioVendor (Brno, Czech Republic).

Table 1 displays the main characteristics of the assays, including the biological matrix, reagents kits, normal ranges, inter-assay imprecision (CV%), and the limits of detection (LOD). The list of the 12 cytokines analyzed together with their sensitivity is reported in Table 2.

**Table 1:** Main characteristics of the analytical methods used for the laboratory tests

Test (unit)	Analyser/kits	Matrix	Reference range	Reproducibility CV%	LOD
BNP (pg/mL)	DxI 800/Biosite	Plasma EDTA	5-100	5.4-6.7	1
NT-proBNP (pg/mL)	Dimension/Siemens	Serum	< 450 (> 75 yrs)	3.9-4.3	NR
TSH (μU/mL)	DxI 800/Beckman	Serum	0.2-3.5	3.72-4.96	0.003
Free T4 (ng/dL)	DxI 800/Beckman	Serum	0.9-1.8	4.3-8.8	0.5
Parathyroid hormone (pg/ml)	DxI 800/Beckman	Serum	16-81	2.8-6.4	1
Creatinine (mg/dL)	DxC 800/Beckman	Serum	0.6-1.4	1.7-9.5	0.05
Cystatin C (mg/L)	DxC 800/Gentian	Serum	0.5-1.0	2.95 - 4.38	<0.1
HDL (mg/dL)	DxC 800/Beckman	Plasma heparin	> 60	2.1-3.4	5
Total cholesterol (mg/dL)	DxC 800/Beckman	Plasma heparin	< 200	1.48-1.6	5
Triglycerides (mg/dL)	DxC 800/Beckman	Plasma heparin	< 150	2.2-2.6	10
Haemoglobin (g/dL)	Sysmex XE-2100	Whole blood	12-17	0.5-1.0	<1.0
Calcium (mg/dL)	DxC 800/Beckman	Serum	8.6-10.0	1.0-2.2	2
Phosphorus (mg/dL)	DxC 800/Beckman	Serum	2.4-4.7	0.6-3.0	0.5
Transthyretin (mg/dL)	DxC 800/Beckman	Serum	18-38	3.6-4.2	2
usCRP (mg/dL)	DxC 800/Beckman	Serum	< 0.3	4.1-5.1	0.02
CMV IgG (A.U./L)	Architect i4000/Abbott	Plasma EDTA	< 6 (normal) 6-15 (suspicion)	5.9-6.2	NA
25-OH-Vitamin D (ng/mL)	Liaison/Diasorin	Serum	30-100	5.2-8.4	< 5.0
IGF1 (ng/mL)	Liaison/Diasorin	Serum	70-140 (> 75 yrs)	5.8-6.1	< 15
Osteocalcin (ng/mL)	Liaison/Diasorin	Serum	3-7	7.4-7.6	< 1.0
Aluminium (μg/dL)	ICP-MS, Agilent/NA	Plasma heparin*	0.2-1.0	8.9	0.1
Copper (μg/dL)	ICP-MS, Agilent/NA	Plasma heparin*	70-170	1.22	3.0
Zinc (μg/dL)	ICP-MS, Agilent/NA	Plasma heparin*	70-120	1.39	5
Selenium (μg/dL)	ICP-MS, Agilent/NA	Plasma heparin*	5-15	1.18	0.5
CC16	ELISA	Serum	2-20	4-9	1.0
SPD	ELISA	Serum	50-150	4.2-9.8	0.2

CV: coefficient of variation; LOD: limit of detection; BNP: brain natriuretic peptide; NT-proBNP: N-terminal pro-brain natriuretic peptide; NR: not reported; TSH: thyroid stimulating hormone; HDL: high-density lipoprotein; usCRP: ultrasensitive C-reactive protein; CMV IgG: cytomegalovirus immunoglobulin G; NA: not applicable; IGF: insulin-like growth factor; * special sampling tubes free of aluminium and other trace elements; CC16: Clara cell protein 16; SPD: surfactant protein D.

**Table 2:** Panel of cytokines measured on the evidence investigator ™ analyzer (Randox) with their respective analytical ranges, imprecision and sensitivities

Cytokine Array I panel	Assay range	Imprecision CV%	LOQ
Interleukin-1 α (IL-1 α) (pg/mL)	0-500	5.9-10.7	0.8
Interleukin-1 β (IL-1 β) (pg/mL)	0-250	7.0-13.1	1.6
Interleukin-2 (IL-2) (pg/mL)	0-3000	7.2-10.0	4.8
Interleukin-4 (IL-4) (pg/mL)	0-900	6.4-10.0	6.6
Interleukin -6 (IL-6) (pg/mL)	0-900	8.7-15.4	1.2
Interleukin-8 (IL-8) (pg/mL)	0-3000	8.7-10.1	7.9
Interleukin-10 (IL-10) (pg/mL)	0-1000	6.1-9.4	1.8
Epidermal Growth Factor (EGF) (pg/mL)	0-900	4.0-7.4	2.9
Interferon-γ (IFN- γ) (pg/mL)	0-1500	9.1-13.4	3.5
Monocyte Chemotactic Protein-1 (MCP-1) (pg/mL)	0-1500	5.6-14.7	13.2
Tumour Necrosis Factor- α (TNF- α) (pg/mL)	0-1500	8.1-13.0	4.4
Vascular Endothelial Growth Factor (VEGF) (pg/mL)	0-3000	6.0-13.4	14.6

CV: coefficient of variation; LOQ: limit of quantification as the lowest concentration with imprecision < 20% for 20 replicates

### Study population

**Figure 1:**
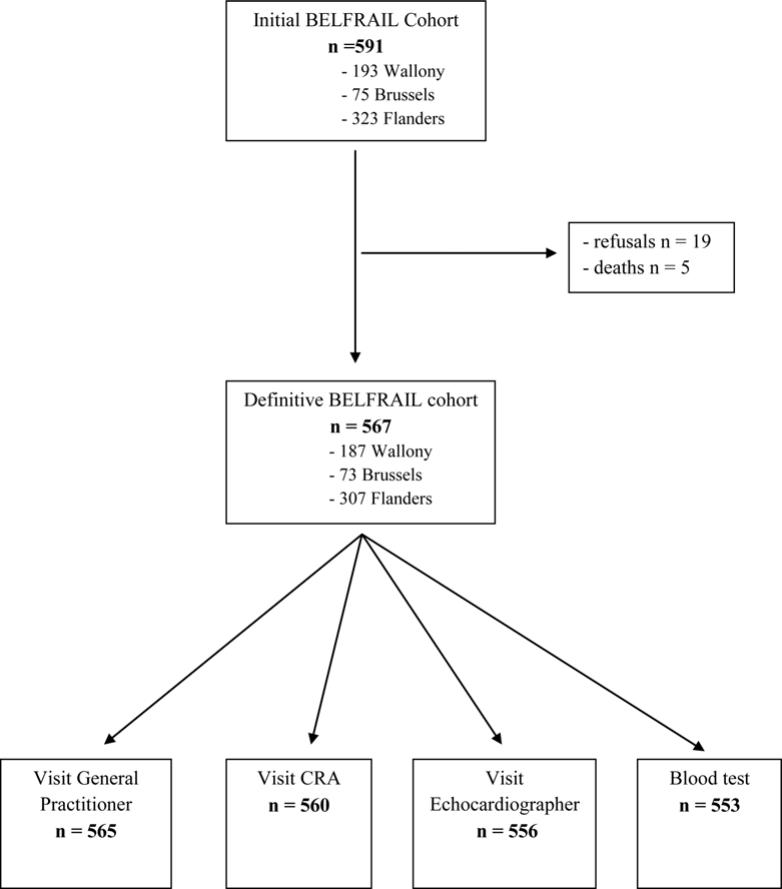
**Inclusion of study participants**.

Between November 2, 2008 and September 15, 2009, 567 subjects were included in the BF_C80+ _(Figure 1). Three hundred subjects were included using the first sampling method and 267 using the second sampling method. All study participants underwent at least one study visit and 546 participants underwent all visits. Table 3 shows the socio-demographic characteristics of the study population. Women represented 63.1% (n = 358) of the study population and had a mean age of 85.0 ± 3.9 years. Male participants had a mean age of 84.3 ± 3.3 years. The majority of study participants lived at home (89.9%) and had a low level of education (69.5% ≤ lower secondary education). Of the participants living at home 188 (36.9%) received professional help and 84 (16.5%) professional care at home.

**Table 3:** Socio-demographic data of participants in the BELFRAIL cohort (n = 567)

	**n (%)**	**Belgian population, %**
	
Male	209 (36.9)	33.5
Age		
Women 80-84	204 (36.0)	36.8
Women 85-89	116 (20.5)	20.6
Women ≥ 90	38 (6.7)	9.2
Men 80-84	130 (22.9)	21.5
Men 85-89	66 (11.6)	9.4
Men ≥ 90	13 (2.3)	2.7
Current Family Situation		
Married	239 (42.5)	
Divorced	11 (2.0)	
Widow(er)	281 (49.9)	
Single	20 (3.6)	
Other	12 (2.1)	
Institutionalized	57 (10.1)	
Level of Education		
Without qualifications	5 (0.9)	
Primary school	207 (36.8)	
Lower secondary education	179 (31.8)	
Higher secondary education	98 (17.4)	
College	55 (9.8)	
University	17 (3.0)	
Professional help at home	188 (36.9)	
Care at home	84 (16.5)	

**Figure 2:**
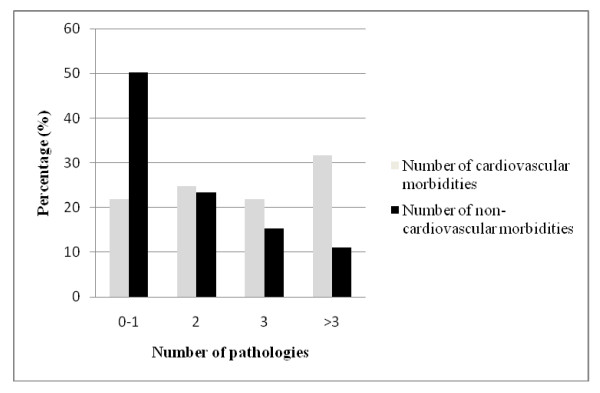
**Number of comorbidities**.

There was a high burden of comorbidity in the population under study. Only six participants (1.1%) were free of any pathology. Non-cardiovascular morbidity was reported in 459 participants (81.2%) (Table 4) and cardiovascular morbidity in 524 participants (92.7%) (Table 5). Figure 2 shows that a high proportion of the study population had three or more non-cardiovascular (26.4%) or cardiovascular (53.5%) pathologies.

**Table 4:** Non-cardiovascular morbidity of participants in the BELFRAIL cohort (n = 567)

	Active pathology* n (%)	Cured pathology n (%)	Unknown^$^, n (%)
Thyroid dysfunction	51 (9.0)	23 (4.1)	1 (0.2)
Hypofunction	47 (8.4)	5 (0.9)	2 (0.4)
Hyperfunction	1 (0.2)	15 (2.7)	3 (0.5)
Anaemia	50 (8.9)	23 (4.1)	0 (0)
Asthma	27 (4.8)	7 (1.2)	5 (0.9)
COPD	65 (11.5)	-	12 (2.1)
Parkinson's disease	16 (2.8)	-	3 (0.5)
Arthritis	28 (5.0)	23 (4.1)	1 (0.2)
Osteoarthritis	324 (58.0)	-	4 (0.7)
Osteoporosis	125 (22.5)	-	17 (3.1)
Cancer	43 (7.6)	73 (12.9)	5 (0.9)
Depression	74 (13.1)	32 (5.7)	8 (1.4)
Renal insufficiency	64 (11.4)	5 (0.9)	9 (1.6)
Hip prosthesis^£^	73 (13.0)	-	-
Knee prosthesis^£^	44 (7.8)	-	-

*Present (even if well controlled by treatment) or having occurred less than six months ago; ^$^Unknown or doubt by general practitioner; ^£^Less or more than 12 months ago.

**Table 5:** Cardiovascular morbidity in participants in the BELFRAIL cohort (n = 567)

	**n (%)**	Objectified*, n (%)	**Unknown^$^, n (%)**
	
Hypertension	396 (70.1)	-	2 (0.4)
Hyperlipidaemia	245 (44.0)	-	3 (0.5)
Diabetes	107 (18.9)	-	4 (0.7)
Angina pectoris	93 (16.5)	58 (62.4)	24 (4.3)
Myocardial infarction	62 (11.0)	59 (95.2)	6 (1.1)
Cardiomyopathy	56 (10.0)	-	-
TIA	58 (10.5)	34 (58.6)	9 (1.6)
CVA	46 (8.3)	40 (87.0)	5 (0.9)
Peripheral arterial disease	52 (9.2)	43 (82.7)	15 (2.7)
Episode of decompensated heart failure	60 (10.6)	47 (78.3)	7 (1.2)
Episode of atrial fibrillation	113 (20.1)	-	5 (0.9)
Chronic atrial fibrillation	58 (10.3)	-	-
Valvular disease	140 (24.9)	135 (96.4)	7 (1.2)
Peripheral oedema	237 (43.8)	-	5 (0.9)

*The general practitioner was asked to report if the pathology was objectified at the time of the diagnosis; ^$^Unknown or doubt by general practitioner.

Table 6 shows the time between the different study visits.

**Table 6:** Time in days between different study visits

	**Median [IQR]**
	
GP-CRA	21 [10-37]
GP-blood test	28 [14-43]
GP-echocardiography	29 [14-43]
CRA-echocardiography	7 [3-15]
CRA-blood test	7 [4-16]
Blood test-echocardiography	3 [1-13]

IQR: interquartile range

Table 7 describes the basic functional status of participants in the BF_C80+_. In general, there was a low prevalence of functional limitations in the population under study, with 9.8% having a score ≤ 15 on the ADL scale and 10% having a score > 10 on the IADL scale. A large number of subjects had a high score on the GDS-15 questionnaire, with 20.6% scoring ≥ 5. Only 15 subjects (2.9%) had an MMSE score ≤ 15. In 8.7% of the study participants, an increased risk of falls according to the Tinetti test was found.

**Table 7:** Functional status of the BF_C80+ _population (n = 567)

	**N**	**Median [IQR]**	**Cut-off value**	**n (%)**
	
ADL	559	25 [21-27]		
GDS-15	557	2 [1-4]	≥ 5	115 (20.6)
MMSE	557	28 [25-29]	≥ 25	443 (79.5)
			21-24	70 (12.6)
			10-20	39 (7.0)
			≤ 9	5 (0.9)
Tinetti	549	27 [24-28]	25-28	406 (74.0)
			19-24	95 (17.3)
			≤ 18	48 (8.7)
IADL	559	6 [6-8]		

ADL: Activities of Daily Living; GDS-15: 15-item Geriatric Depression Scale; MMSE: Mini-Mental State Examination; IADL: Instrumental Activities of Daily Living; IQR: interquartile range.

### Follow-up

In October 2009 a basic report for every included patient was sent back to inform the GP. This report included the measurements of height and weight, blood pressure measurements, the results of the performance tests, ADL, MMSE, GDS-15, Barthel's Index and Tinetti test, the unvalidated electronically generated results of the spirometry and ECG and a basic report on major cardiac abnormalities found with echocardiography.

After a follow-up period of a maximum 16 months (March 2010 for Wallony and July 2010 for Flanders and Brussels), and yearly thereafter, a detailed follow-up questionnaire will be sent to the participating GP centres. The GPs will be asked to fill out a questionnaire for every included patient describing his/her health status. The questionnaire includes questions on mortality and cause of mortality, episodes (> 1 day) and cause of hospitalization, change of living and family situation and new comorbidities (fall, cardiovascular event, heart failure, new diagnosis of cancer, renal replacement therapy) that occurred in the past 16 months. The GPs will be asked to report changes in treatment and to record if they changed the management of the patient based on the basic report that was sent back. For every included patient, the GP will be asked if he agrees that the patient can be contacted for the follow-up examination.

In May 2010 (Wallony) and September 2010 (Flanders and Brussels) every participant will be contacted again for the follow-up examination. The follow-up examination will include anthropometric measurements, evaluation of blood pressure, vision, hearing, incontinence, anamnesis, performance testing, ADL, GDS-15, MMSE, Tinetti Test, GFI, LOC, grip strength, spirometry, ECG, bio-electrical impedance, exhaled NO, echocardiography and new blood tests.

### Data check and analysis

Data entry of all questionnaires and examination forms is done by the data manager. Data is inserted with FileMaker^® ^Pro 8.0 (FileMaker Inc., Santa Clara, CA, USA) or Microsoft Excel^®^. The quality of data entry is systematically verified by a trained researcher in order to detect errors. SPSS 16.0 (SPSS Inc., Chicago, IL, USA) will be used for data analysis. Analyses will combine cross-sectional and prospective approaches. The prospective analyses will be performed on the entire cohort. Outcome measures will be registered for every patient that underwent at least one module of the assessment. The variety of dimensions included in the BF_C80+ _will enable us to correct for a wide range of factors in the analyses and multivariate models.

For the cross-sectional part of the study, very few data are missing. For most variables, less than 5% of data are missing. Only the following variables have more missing data: calf skin fold (n = 55, 9.7%), sense of coherence (n = 70, 12.3%), locus of control (n = 71, 12.5%), bio-electrical impedance (n = 278, 49.0%) and exhaled NO (n = 311, 54.9%).

Future analysis and publications from this cohort will be carried out using the STROBE criteria [67].

## Discussion

In the BELFRAIL Cohort Study we were able to include 567 participants who underwent a thorough multi-dimension investigation in order to take a detailed "medical photograph" of every included subject. We were able to construct a cohort representative in gender and age of the very elderly living in Belgium. The mean age of women and men older than 80 living in Belgium (84.9 years and 83.9 years, respectively) [68] was comparable to the mean age found in this cohort. Moreover, the fact that participants were included by their GP enabled us to create a heterogeneous population representative of the very elderly since more than 90% of people aged 80+ in Belgium regularly see their GP.

This is the first cohort study of very elderly people living in Belgium. To our knowledge this is also the first cohort of elderly in which a large set of technical examinations (ECG, spirometry, echocardiography, bio-electrical impedance, etc.) was done at home or in a nursing home. People aged 80+ are the fastest growing segment of the population in industrialized countries, and their number will peak in 2050. Research trying to understand the complexity of the geriatric patient is needed in order to anticipate booming health care expenditures and guarantee future fundable social security. Therefore, the BF_C80+ _was designed to acquire a better understanding of the epidemiology and pathophysiology of chronic diseases in the very elderly, and to study the dynamic interaction between health, frailty and disability in a multi-system approach.

The BF_C80+ _focuses on cardiac dysfunction and chronic heart failure, lung function, sarcopenia, renal insufficiency and immunosenescence. This wide variety of dimensions will enable us not only to investigate in depth the relation between these different systems but also to instigate new research questions with this unique database of community-dwelling elderly.

## List of abbreviations used

GP: general practitioner; CRA: clinical research assistant; BNP: brain natriuretic peptide; NT-proBNP: N-terminal pro-brain natriuretic peptide; ECG: electrocardiogram; COPD: chronic obstructive pulmonary disease; CKD: chronic kidney disease; GFR: glomerular filtration rate; CMV: cytomegalovirus; UCL: Université Catholique de Louvain; CD: compact disc; MMSE: Mini-mental state examination; TIA: transient ischaemic attack; CVA: cerebro-vascular accident; PTCA: percutaneous transluminal coronary angioplasty; MRC: Medical Research Council; JVP: jugular venous pressure; NYHA: New York Heart Association; ADL: activities of daily living; LAPAQ: LASA physical activity questionnaire; GDS: geriatric depression scale; IADL-E: instrumental activities of daily living; IADL: index of activities of daily living; GFI: Groningen frailty indicator; SOC: sense of coherence; LOC: locus of control; MHLC: multidimensional health locus of control; FVC: forced vital capacity; ISO: International Organization for Standardization; FDA: Food and Drug Administration; BIA: bioelectrical impedance analysis; FFM: fat-free mass; SMM: skeletal muscle mass; eNO: exhaled nitric oxide; 2D: two-dimensional; LV: left ventricular; TSH: thyroid stimulating hormone; HDL: high-density lipoprotein; LDL: low-density lipoprotein; usCRP: ultrasensitive C-reactive protein; CMV IgG: cytomegalovirus-specific immunoglobulin G; IGF: insulin-like growth factor; CC16: Clara cell protein 16; SPD: surfactant protein D; CV: coefficient of variation; LOD: limit of detection; STROBE: Strengthening The Reporting of OBservational studies in Epidemiology.

## Competing interests

The authors declare that they have no competing interests.

## Authors' contributions

BV, principal investigator, drafted the manuscript. JD and BV initiated the BELFRAIL study, and are responsible for its design, conduct and analysis. As members of the BELFRAIL study committee, AP and PW are involved in the development of the study and obtaining research funding. NR performed all echocardiography, and NR and AP are responsible for the analyses of the echocardiography. PW, HM and PAO are responsible for the laboratory analyses. All authors participated in the critical revision of the manuscript.

## Pre-publication history

The pre-publication history for this paper can be accessed here:

http://www.biomedcentral.com/1471-2318/10/39/prepub
